# Spine Formation Pattern of Adult-Born Neurons Is Differentially Modulated by the Induction Timing and Location of Hippocampal Plasticity

**DOI:** 10.1371/journal.pone.0045270

**Published:** 2012-09-14

**Authors:** Noriaki Ohkawa, Yoshito Saitoh, Eri Tokunaga, Itsuko Nihonmatsu, Fumiko Ozawa, Akiko Murayama, Fumi Shibata, Toshio Kitamura, Kaoru Inokuchi

**Affiliations:** 1 Department of Biochemistry, Graduate School of Medicine and Pharmaceutical Sciences, University of Toyama, Sugitani, Toyama, Japan; 2 JST, CREST, Kawaguchi, Japan; 3 Mitsubishi Kagaku Institute of Life Sciences, MITILS, Machida, Tokyo, Japan; 4 Division of Cellular Therapy, Institute of Medical Science, University of Tokyo, Minato-ku, Tokyo, Japan; University of Nebraska Medical Center, United States of America

## Abstract

In the adult hippocampus dentate gyrus (DG), newly born neurons are functionally integrated into existing circuits and play important roles in hippocampus-dependent memory. However, it remains unclear how neural plasticity regulates the integration pattern of new neurons into preexisting circuits. Because dendritic spines are major postsynaptic sites for excitatory inputs, spines of new neurons were visualized by retrovirus-mediated labeling to evaluate integration. Long-term potentiation (LTP) was induced at 12, 16, or 21 days postinfection (dpi), at which time new neurons have no, few, or many spines, respectively. The spine expression patterns were investigated at one or two weeks after LTP induction. Induction at 12 dpi increased later spinogenesis, although the new neurons at 12 dpi didn’t respond to the stimulus for LTP induction. Induction at 21 dpi transiently mediated spine enlargement. Surprisingly, LTP induction at 16 dpi reduced the spine density of new neurons. All LTP-mediated changes specifically appeared within the LTP–induced layer. Therefore, neural plasticity differentially regulates the integration of new neurons into the activated circuit, dependent on their developmental stage. Consequently, new neurons at different developmental stages may play distinct roles in processing the acquired information by modulating the connectivity of activated circuits via their integration.

## Introduction

Many works have clarified that new neurons are continually being generated in the adult hippocampus (HP) of different mammalian species including monkey and human [1], although most results have been proposed from model animals, especially rodents. The dentate gyrus (DG) of the HP integrates newly born neurons throughout adult life. Adult-born neurons are required for various types of brain function including HP-dependent memory [2]–[5]. Numerous factors associated with various behavioral and cognitive states of animals regulate adult neurogenesis in the DG, and HP-dependent learning is one of the major regulators of this neurogenesis [5]. Learning of HP-dependent tasks, but not HP-independent tasks, enhances neurogenesis in DG [5], [6]. In addition, HP-dependent learning has been suggested to selectively add and remove new neurons according to their maturity and functional relevance [7], [8].

In the central nervous system, postsynaptic spines on neuronal dendrites interact with presynaptic axonal terminals, and the majority of glutamatergic excitatory inputs are received by the dendritic spines of postsynaptic neurons [9]. The dendritic spine is a critical site for synaptic plasticity. Therefore, spine expression pattern is one indicator of the integration of neurons into excitatory synapses. Retrovirus (RV)-based gene transfer is a useful methodology to mark progenitor cells and their progeny in the DG because the viral genome is only integrated into proliferating cells [10]. Therefore, green fluorescence protein (GFP)-RV has been used to visualize the morphology of newly born neurons derived from DG progenitor cells [11]. GFP-RV-mediated labeling has revealed distinct morphological stages of newly born neurons during development [11]. Their spine expression pattern is closely correlated with the excitatory postsynaptic response of developing newly born neurons in adult DG [11]–[14].

Spatial learning is dependent on HP and influences neurogenesis in adult DG [6]–[8]. Notably, training in spatial learning by water maze during new neurons’ second week of age enhances the complexity of their dendritic arbor and spine formation rate [15]. Thus, new neurons pass through several developmental stages, during which each neuron could be differentially integrated into circuits in an experience-dependent manner. However, it remains unclear whether the later synaptic integration pattern of new neurons is differentially modulated by the timing of experiences, and whether the integration is regulated locally in a synaptic input-specific manner or as a cell-wide event.

**Figure 1:**
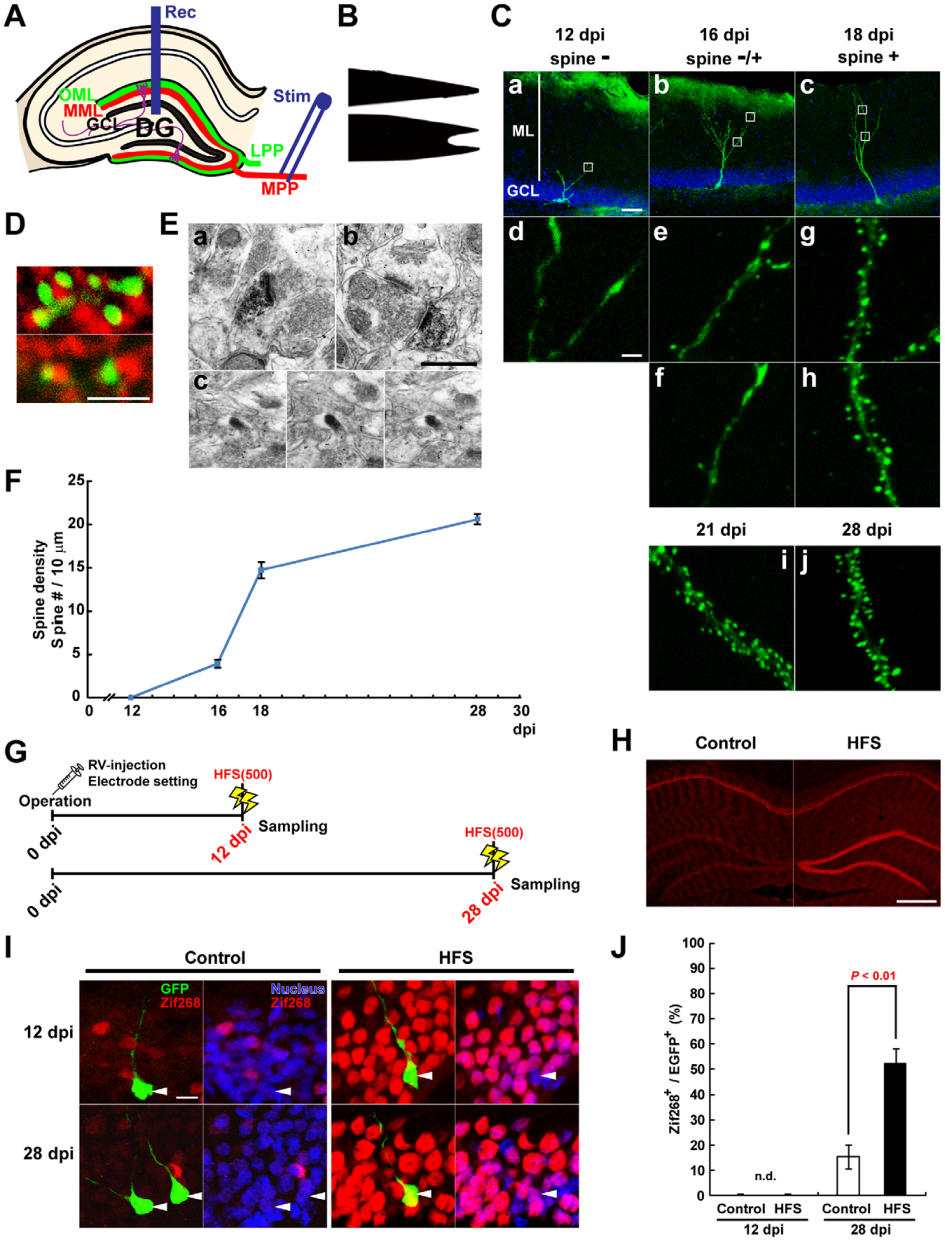
Spinogenesis of new neurons during the first 4 weeks after birth in adult DG. (**A**) Anatomical organization of the entorhinal-hippocampal DG pathway. Abbreviations: Rec, recording electrode; Stim, stimulating electrode; MPP and LPP, medial and lateral perforant pathway, respectively; MML and OML, middle and outer molecular layer, respectively; GCL, granule cell layer. (**B**) Side views photos showing needle tip, shaped by electrical grinder, of Hamilton syringes used for RV injection, at 90 degree orientations with respect to each other. (**C**) Representative z-stack images of morphologies and dendritic segments from newly born neurons at 12, 16, 18, 21, and 28 dpi. New neurons were identified by RV-mediated labeling with GFP-actin (green). Blue indicates nuclear distribution of individual cells (DRAQ5 staining). ML, molecular layer; GCL, granule cell layer. Selected regions in low magnification images (within squares) at 12, 16, and 18 dpi are shown below in high magnification. Scale bars: **a-c**, 50 µm; **d**, 5 µm; **e-j**, 2 µm. (**D**) Spines of new neurons are contacted by presynaptic terminals. Z-stack images are observations of dendritic fragments from discrete cells at 21 dpi. GFP-actin signal allows visualization of spines (green). The presynaptic marker synaptophysin is shown in red. Nearly all spines labeled with GFP-actin contact presynaptic terminals. Scale bar, 2 µm. (**E**) Immunoelectron microscopy showed synapse formation of GFP-actin-positive spines on 28 dpi neurons as defined by containing postsynaptic density and contacting with synaptic vesicles containing structure. **a** and **b**, typical images of dendritic spines of new neurons. **c**, Images of a filopodial protrusion were taken by tilting function (left, middle, and right photo: −50°, 0°, and +40°, respectively). Scale bar, 0.5 µm. (**F**) Density of dendritic spines on new neurons labeled with GFP-actin-RV. Line graph shows the density of protrusions on dendritic fragments of new neurons at 12, 16, 18, and 28 dpi. The protrusions density is expressed as number of protrusions per 10-µm dendritic length. (**G-J**) HFS(500)-mediated Zif268 expression in new neurons at 12 and 28 dpi. (**G**) Experimental schedule. HFS(500) was unilaterally delivered to MPP, and brains were dissected at 1 h after the initiation of HFS. (**H**) Immunohistochemistry with anti-Zif268 antibody. Left, HP of control hemisphere; right, ipsilateral hemisphere treated with HFS. Scale bar, 0.5 mm. (**I**) Triple staining for Zif268 (red), GFP (green), and DAPI (nucleus, blue). Arrowheads in GFP/Zif268 and Nucleus/Zif268 photos in each panel indicate the soma of the same GFP-labeled new neurons. Scale bar, 10 µm. (**J**) Percentage of Zif268-positive new neurons in control and HFS-delivered DG. At 12 dpi, Zif268^+^ and GFP^+^ double-positive cells were not detected (n.d.). At 12 dpi, n = 3 animals; control hemisphere, n = 15, 12, and 17 GFP+ cells in each animal; HFS-delivered hemisphere, n = 13, 12, and 7 GFP+ cells in each animal. At 28 dpi, n = 3 animals; control hemisphere, n = 19, 4, and 18 GFP+ cells in each animal; HFS-delivered hemisphere, n = 22, 8, and 15 GFP+ cells in each animal. *P* values from Student’s *t*-test are shown in the graph.

Long-term potentiation (LTP) is a designated model of activity-dependent synaptic plasticity. One type of learning has been reported to induce LTP in HP and produce the same change in mechanisms underlying the LTP induction of excitatory synapses [16]. Therefore, LTP is now widely accepted as a critical component of neural mechanisms underlying learning and memory [17], [18]. DG LTP induction in freely moving animals has the advantage of allowing the long-lasting effects of synaptic plasticity induction on various phenomena to be observed. In DG, each neuron consisting of a granule cell (GC) receives two inputs from the entorhinal cortex, via the medial and lateral perforant path (MPP and LPP, respectively). MPP and LPP share the lamination domain of the molecular layer (ML), comprising its middle third (MML) and its outer third (OML), respectively [19]–[21] (Figure 1A). Newly born neurons also form functional synapses with the MPP and LPP [22], [23].

High-frequency stimulation (HFS) of MPP or LPP fibers specifically results in LTP at the MML or OML synapses, respectively [21]. Establishment of layer (input)-specific long lasting late LTP (L-LTP) is mediated by a synaptic tagging mechanism underlying the input-specific function of proteins at activated synapses that are newly synthesized at soma [24], [25].

In this paper, we address the questions of whether the LTP induction at new neurons’ different ages affects later spine expression patterns in an induction-timing-dependent manner, and whether these effects are restricted to the area where LTP is induced or if it is spread cell-wide. Our results indicate timing-dependent and area-specific regulation of spine expression patterns.

## Methods

### Reagents and Antibodies

The primary antibodies were from the following sources: rabbit anti-GFP antibody, Invitrogen (Carlsbad, CA); chicken anti-GFP antibody, Abcam (Cambridge, MA); mouse anti-synaptophysin antibody, Prof. M. Takahashi, Kitasato University, Japan; rabbit anti-Egr-1 (Zif268), Santa Cruz Biotechnology, Inc (Santa Cruz, CA); rat anti-bromodeoxyuridine (BrdU) antibody, Serotech (Raleigh, NC); and mouse anti-NeuN antibody, Chemicon (Temecula, CA). Fluorescein (FITC)- and rhodamine-conjugated donkey anti-rabbit secondary antibodies were purchased from Chemicon. FITC-conjugated donkey anti-chick secondary antibody was from Jackson ImmunoResearch (West Grove, PA). Alexa Fluro-546-conjugated goat anti-mouse, Alexa Fluro-488-conjugated donkey anti-rat, Alexa Fluro-546-conjugated goat anti-rabbit, and Alexa Fluro405-conjugated goat anti-mouse IgG antibodies were from Invitrogen (Carlsbad, CA). DRAQ5 (Biostatus Ltd, Leicestershire, UK), which fluoresces in the far red, or DAPI (Roche, Mannheim, Germany) were used for nuclear staining. Phalloidin-tetramethylrhodamine B isothiocyanate (TRITC) (Sigma-Aldrich, MO) was used for F-actin staining. CPP was purchased from Tocris Coolson Inc. (Bristol, UK).

### Animals

All procedures involving the use of animals complied with the guidelines of the National Institute of Health and were approved by the Animal Care and Use Committees of Mitsubishi Kagaku Institute of Life Sciences and University of Toyama. Male Wistar ST rats (Japan SLC Inc., Shizuoka, Japan) approximately 20 weeks of age were used for retroviral labeling and LTP experiments.

### Retrovirus Production

The pMXs-SIN-CAG-GFP was developed from RV vector pMXs [26]. First, a PvuI-SacI fragment was deleted from pMXs, followed by blunting the SacI site using a Blunting Kit (Takara, Japan), and ligating PvuI and the blunted SacI. The resulting vector pMXs-SIN (self-inactivating) was linearized by PacI in the 5′-multi-cloning site and blunted as above, where the CAG promoter was inserted. Finally, a GFP cDNA was inserted downstream of the CAG promoter using the EcoRI and NotI sites.

The pMXs-SIN-CAG-GFP-β-actin was prepared as follows. Rat β-actin cDNA prepared from rat PC-12 cell mRNAs was kindly donated by Prof. M. Takahashi, Kitasato University, Japan. The β-actin open reading frame cDNA was subcloned into EcoRI and BamHI sites of pEGFP-C2 (Clontech, Mountain View, CA). The EGFP-β-actin fragment was generated by Eco47III and BamHI digestion, and then replaced with the GFP fragment of the pMXs-SIN-CAG-GFP vector. The pMXs-SIN-CAG-GFP or -GFP-β-actin was co-transfected with pVSV-G (Clontech) into PLAT-gp cells, counterparts of PLAT-E cells lacking the transgene for envelope [27], by FuGENE HD (Roche Applied Science, Indianapolis, IN). RV-containing culture supernatants were collected after 2 days, and the RV was concentrated by ultracentrifuge at 5×10^4^×*g* for 90 min at 4°C. The RV pellet was resuspended to 0.5% of original volume in phosphate-buffered saline (PBS).

### Labeling of New Neurons

For high accuracy injection of RV solution into DG, the needle tip of a Hamilton syringe was shaped as shown in Figure 1B using an electrical grinder. Rats were anesthetized with pentobarbital (55 mg/kg, i.p. injection), and 3 µl per hemisphere of RV solution was infused bilaterally into the DG positioned 3.6 mm posterior; ±1.9 mm lateral, and 4.2 mm ventral to bregma (0.5 µl/min).

Procedure to label newly born cell with bromodeoxyuridine (BrdU) was described previously [4]. Briefly, rats were injected with BrdU in 0.9% NaCl solution (100 mg/kg, i.p., Sigma) twice a day (6 h interval) for three consecutive days, and were perfused 27 days later the last injection.

### DG LTP in Unanesthetized Freely Moving Animals

The surgical procedure was as described previously [21]. After the RV injection procedure, the electrode stimulating the MPP fibers was positioned 8.7 mm posterior, 5.3 mm lateral, and 5.3 mm ventral to bregma. A recording electrode was implanted ipsilaterally 4.0 mm posterior, 2.5 mm lateral, and 3.8 mm ventral to bregma. Electrode placement was only performed on the right ( = ipsilateral) hemisphere.

LTP experiments on freely moving animals were performed as described previously [21], [28]. LTP was induced by tetanic stimuli with biphasic square waveform, 200-µs pulse width. Maximal population spike (PS) amplitude was determined, and the intensity of the stimulus current was set to elicit 60% of the maximal PS amplitude. This intensity was used for baseline recording and HFS(500) experiments. The animals were transferred to the recording chamber, and the baseline response was monitored by delivering test pulses (0.05 Hz) for 15 min. After the baseline monitoring, LTP was induced by high frequency tetanic stimulation, HFS(500), consisting of 10 trains with 1-min intertrain intervals. Each train consisted of five bursts of 10 pulses at 400 Hz, delivered at 1-s interburst intervals. Synaptic transmission was monitored for 15 min immediately, 1 day, 7 days, 12 days, and 16 days after HFS(500) stimulation. The local induction at MML in each animal was confirmed by F-actin staining.

In CPP experiments, the baseline monitoring was performed 1 day before HFS(500). CPP was dissolved in saline (0.9% saline), and the rats were injected i.p. with 10 mg/kg CPP 2 h before the beginning of HFS(500) stimulation. Synaptic transmission was monitored for 15 min at 1 day and 7 days after HFS(500) stimulation.

### Histochemistry

Rats were deeply anesthetized with an overdose of pentobarbital solution and perfused transcardially with PBS, pH 7.4, followed by 4% paraformaldehyde (PFA) in PBS. The brains were removed and further post-fixed by immersion in 4% PFA in PBS for 2 h at 4°C. Each brain was equilibrated in 25% sucrose in PBS, then frozen in dry-ice powder. For F-actin staining, coronal sections (14-µm thickness) were cut on a cryostat and washed with PBS. The sections were incubated with phalloidin-TRITC (0.1 ng/ml) at 4°C for 24 h. The sections were treated with DAPI (1 µg/ml) or DRAQ5 (1∶5000) and then washed with PBS three times, 10 min/wash. For GFP staining, coronal sections were cut on a cryostat at 50-µm thickness, 3.1 mm to 4.9 mm from bregma (36 sections total) and transferred to 12-well cell culture plates (Corning, Corning, NY) containing PBS. After washing with PBS, the floating sections were treated with PBST (PBS supplemented with 0.5% Triton X-100) at room temperature (RT) for 20 min, followed by two 10-min washes with PBS. The sections were then treated with blocking buffer (3% bovine serum albumin in PBS) at RT for 1 h. Reactions with primary antibodies were performed in blocking buffer containing rabbit anti-GFP (1∶1000) and mouse anti-synaptophysin (1∶5000) antibodies at 4°C overnight. After three 10-min washes with PBS, the sections were incubated with FITC-conjugated anti-rabbit and AlexaFluor 546-conjugated anti-mouse IgG secondary antibodies at RT for 3 h. Sections were treated with DAPI (1 µg/ml) or DRAQ5 (1∶5000) and then washed with PBS three times, 10 min/wash.

For BrdU staining, coronal sections were cut on a cryostat, and every other 20 µm coronal section was collected from 3.0 mm to 5.0 mm from bregma (50 sections total). The sections were boiled with 0.01 M sodium citrate buffer (pH 6.0) for 10 min, treated with 2 M HCl for 30 min, and rinsed in 0.1 M boric acid (pH 8.5) for 10 min, as described previously [29]. Sections were blocked with 5% donkey serum in PBS containing 0.1% Triton X-100 at room temperature for 1 h. After blocking, sections were incubated with blocking solution containing rat anti-BrdU (1∶800), rabbit anti-Egr-1 (Zif268) (1∶500), and mouse anti-NeuN (1∶300) antibodies. After washing with PBS, sections were incubated with anti-rat IgG-AlexaFluor 488 (1∶200), anti-rabbit IgG-AlexaFluor 546 (1∶200), and anti-mouse IgG-AlexaFluor 405 (1∶200) antibodies at RT for 3 h. The slides were then washed with PBS three times for 10 min per wash.

Mounting of sections on slide glasses was performed with ProLong Gold antifade reagents (Invitrogen). The fluorescent signals were examined with a laser-scanning confocal microscope (LSM5 PASCAL or LSM700 ZEN, Carl Zeiss, Jena, Germany).

### Immunoelectron Microscopy

Rats were deeply anesthetized with an overdose of pentobarbital solution and transcardially perfused with 4% PFA and 0.05% glutaraldehyde in PBS. The brains were postfixed for 48 h in 4% PFA, equilibrated in 25% sucrose in PBS, then 50-µm coronal vibratome sections were cut. The sections were cryoprotected in 30% sucrose in 0.1 M phosphate buffer (PB) for 2 h and freeze-thawed two times in liquid nitrogen. Immunostaining was performed with an avidin-biotin immunoperoxidase using a VECTASTEIN Elite ABC kit (Vector Laboratories, Burlingame, CA). The sections were then treated with blocking buffer (3 drops of normal goat serum solution of VECTASTEIN Elite ABC kit in 10 ml PBS) at RT for 1 h. Reactions with primary antibodies were performed in blocking buffer containing rabbit anti-GFP (1∶1000) antibody at 4°C overnight. After washing in PBS, the sections were incubated for 3 h at RT in biotinylated secondary antibody (1 drop of goat antibody to rabbit IgG of VECTASTEIN Elite ABC kit in 10 ml blocking solution). To reveal this labeling, we incubated sections at 4°C overnight in avidin-biotin peroxidase complex (VECTASTEIN Elite ABC kit). After washing in 0.1 M PB, the sections were visualized with the 3,3′-diaminobenzidine tetrachloride with nickel (DAB substrate kit, Vector Laboratories) for 5 min. The sections were then postfixed with 1% glutaraldehyde in 0.1 M PB for 10 min, washed in 0.1 M PB, postfixed with osmium tetroxide in 0.1 M PB for 1 h, washed in water, and then treated with 5% uranyl acetate in 50% ethanol for 30 min. The sections were dehydrated by passing through a graded series of ethanol and propylene oxide, and embedded in Epon 812. Ultra thin sections were cut (about 150 nm thick) and observed by tilting function of an electron microscope (100 kV) (JEM-1400, JEOL, Tokyo, Japan).

### Data Analysis

Quantitative measurements of F-actin levels and synaptophysin expression were defined by average signal intensity using Metamorph Software (Molecular Devices, Downingtown, PA).

**Figure 2:**
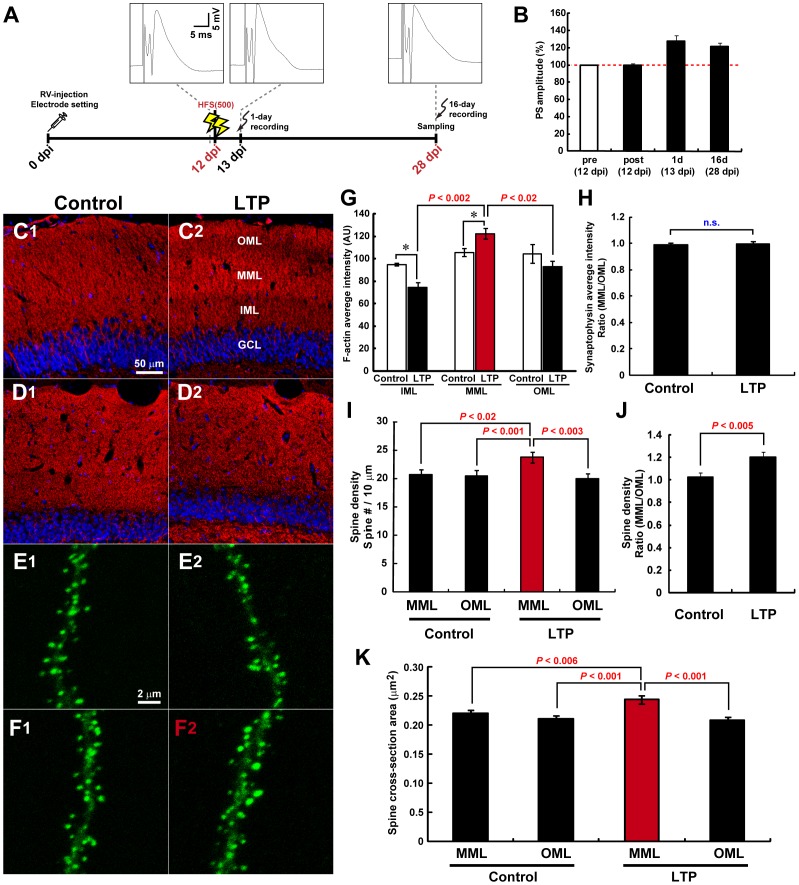
MML-LTP induction at 12 dpi enhances the later spinogenesis of new neurons specifically in MML. (**A**) Experimental schedules for Figure 2. Insets are samples of evoked field potential traces that are recorded at pre-HFS, 1 day, and 16 days post-HFS. (**B**) PS amplitudes of the DG obtained from rats used for experiments in Figure 2. Pre- and post-HFS delivery are indicated by “pre” and “post”, respectively. (**C**) LTP induction changes F-actin content in the DG ML. Unilateral HFS(500) was delivered to the MPP at 12 dpi, and brains were dissected at 28 dpi. DG of control hemisphere (**C1**) and LTP hemisphere (**C2**). F-actin signal (red) was visualized by phalloidin-tetramethyl rhodamine iso-thiocyanate (TRITC) staining. DG subregions are indicated in (**C2**). IML, inner ML. (**D**) Presynaptic content identified by synaptophysin signal are unchanged by MML LTP induction. Fluorescence micrographs with synaptophysin (red) in control (**D1**) and LTP (**D2**) DG. Nuclear signal is shown in blue (DRAQ5). Scale bars for (**C**) and (**D**), 50 µm. (**E, F** *)* Representative z-stack images of dendritic segments of new neurons at 28 dpi in control (**E1, F1**) and LTP hemispheres (**E2, F2**). New neurons were visualized with GFP-actin. (**E1, 2**) and (**F1, 2**) represent micrographs of OML and MML, respectively. Therefore, a dendritic segment within the LTP-induced layer is depicted in (**F2**) only, indicated by red characters. Scale bar, 2 µm. (**G**) F-actin content significantly increases in MML compared with ipsilateral IML and OML and contralateral MML. Graphs show average intensity of F-actin in each DG layer in arbitrary units (AU). *, *P*<0.05 from Student’s t-test. (**H**) Synaptophysin expression is unchanged by MML LTP. Graph shows MML-to-OML ratio (MML/OML) of average synaptophysin intensity in control and LTP hemispheres. n.s. indicates no significant difference. (**I**) Spine density within the LTP-induced layer significantly increases compared with other layers. Spine number per 10-µm dendritic fragment is shown in the graph. (**J**) The graph shows MML-to-OML ratio (MML/OML) of spine density in control and LTP hemispheres, with *P* values from Student’s t-test. (**K**) MML LTP induction enlarges spines in the MML. Average cross-sectional area of spines, an indicator of spine size, is graphed. (**G–K**) Dendritic fragments for spine analyses: control hemisphere, n = 22, LTP hemisphere, n = 22 from 3 animals. Data from the LTP-induced layer are indicated by red color in each graph. *P* values from post-hoc Fisher’s and Scheffe’s test are shown in (**I**), (**K**) and (**G**), respectively.

**Figure 3:**
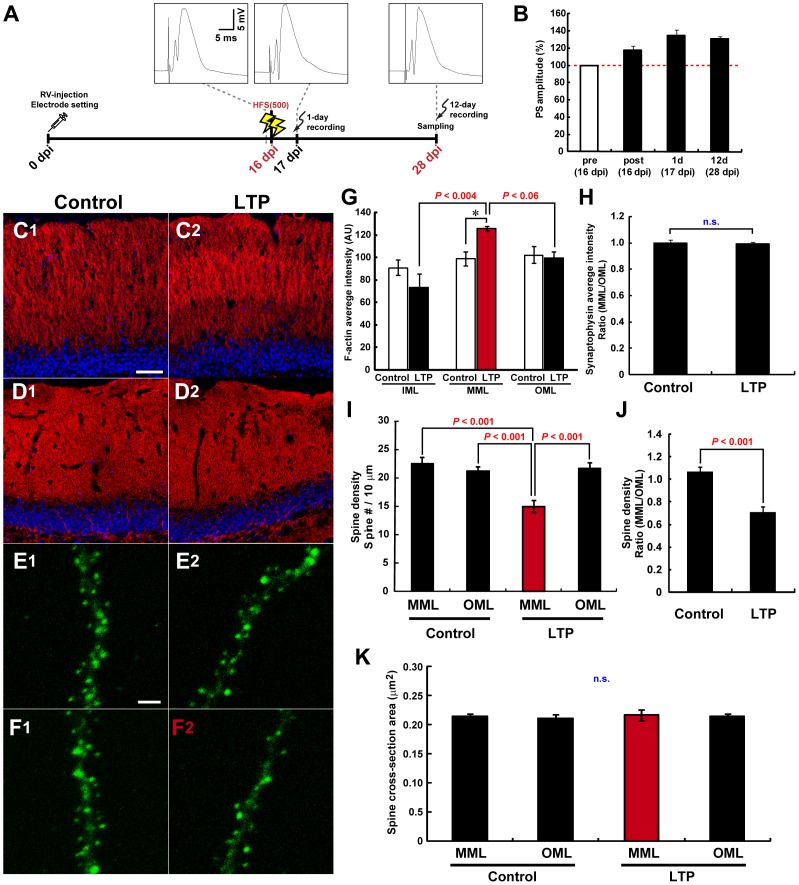
MML-LTP induction at 16 dpi inhibits the later spinogenesis of new neurons specifically in MML. (**A**) Experimental schedules for Figure 3. Insets are samples of evoked field potential traces that are recorded at pre-HFS, 1 day, and 12 days post-HFS. (**B**) PS amplitudes of the DG obtained from rats used for experiments in Figure 3. Pre- and post-HFS delivery are indicated by “pre” and “post”, respectively. (**C**) Fluorescence micrographs of F-actin signal (phalloidin-TRITC, red) in control (**C1**) and LTP-induced DG (**C2**) of 28-dpi animals. Nuclear signal is shown in blue (DRAQ5). (**D**) Synaptophysin (red) and nuclei (DRAQ5, blue) signals of control (**D1**) and LTP hemispheres (**D2**). Scale bars for (**C**) and (**D**), 50 µm. (**E, F**) Representative z-stack images of dendritic segments of new neurons at 28 dpi in control (**E1, F1**) and LTP hemispheres (**E2, F2**). (**E1, 2**) and (**F1, 2**) represent micrographs of OML and MML, respectively. Only (**F2**) represents a dendritic segment within the LTP-induced layer, indicated by red characters. Scale bar, 2 µm. (**G**) Graphs of average F-actin intensity in each DG layer in arbitrary units (AU). *, *P*<0.02 from Student’s t-test. (**H**) Graph shows the MML-to-OML ratio (MML/OML) of average synaptophysin intensity. (**I**) Spine density within the LTP-induced layer significantly decreases compared with other layers. Spine number per 10-µm dendritic fragment in each layer is graphed. (**J**) Graph shows MML-to-OML ratio (MML/OML) of spine density in control and LTP hemispheres. (**K**) Average spine cross-sectional area is indicated. (**G, K**) Dendritic fragments for spine analyses: control hemisphere, n = 18, LTP hemisphere, n = 18 from 3 animals. Data from the LTP-induced layer are indicated by red color in each graph. n.s. indicates no significant difference or variance. *P* values from post-hoc Fisher’s test, Scheffe’s test, and Student’s t-test are shown in (**G**), (**I**), and (**J**), respectively.

**Figure 4:**
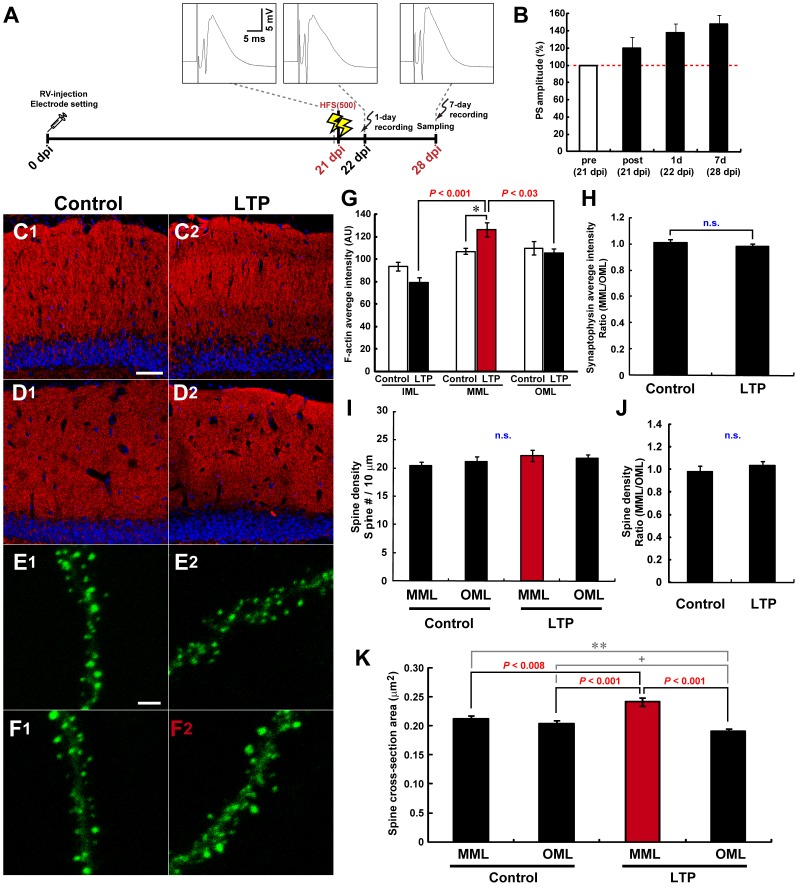
MML-LTP induction at 21 dpi mediates only spine enlargement of new neurons specifically in MML. (**A**) Experimental schedules for Figure 4. Insets are samples of evoked field potential traces that are recorded at pre-HFS, 1 day, and 7 days post-HFS. (**B**) PS amplitudes of the DG obtained from rats used for experiments in Figure 4. Pre- and post-HFS delivery are indicated by “pre” and “post”, respectively. (**C**) Fluorescence micrographs of F-actin signal (phalloidin-TRITC, red) in control (**C1**) and LTP-induced DG (**C2**) of 28-dpi rats. Nuclear signal is shown in blue (DRAQ5). (**D**) Synaptophysin (red) and nucleus (DRAQ5, blue) signals of control (**D1**) and LTP-induced hemispheres (**D2**). Scale bars for (**C**) and (**D**), 50 µm. (**E, F**) Representative z-stack images of dendritic segments of new neurons at 28 dpi in control (**E1, F1**) and LTP hemispheres (**E2, F2**). (**E1, 2**) and (**F1, 2**) represent micrographs of OML and MML, respectively. A dendritic segment within the LTP-induced layer is shown in (**F2**) only, indicated by red characters. Scale bar, 2 µm. (**G**) Graphs of average F-actin intensity in each DG layer in arbitrary units (AU). *, *P*<0.05 from Student’s t-test. (**H**) Graph shows MML-to-OML ratio (MML/OML) of average synaptophysin intensity. (**I**) Spine number per 10-µm dendritic fragment in each layer is graphed. (**J**) Graph shows MML-to-OML ratio (MML/OML) of spine density in control and LTP hemispheres. (**K**) LTP induction enlarges spines expressed within the LTP-induced layer. Average cross-sectional area of spines is indicated. ^+^, *P*<0.063 from Student’s t-test; **, P<0.011 from Mann-Whitney U-test. (**G–K**) Dendritic fragments for spine analyses: control hemisphere, n = 16, LTP hemisphere, n = 16 from 3 animals. Data from the LTP-induced layer are indicated by red color in each graph. n.s. indicates no significant difference or variance. *P* values from post-hoc Fisher’s and Scheffe’s test are shown in (**G**) and (**K**), respectively.

**Figure 5:**
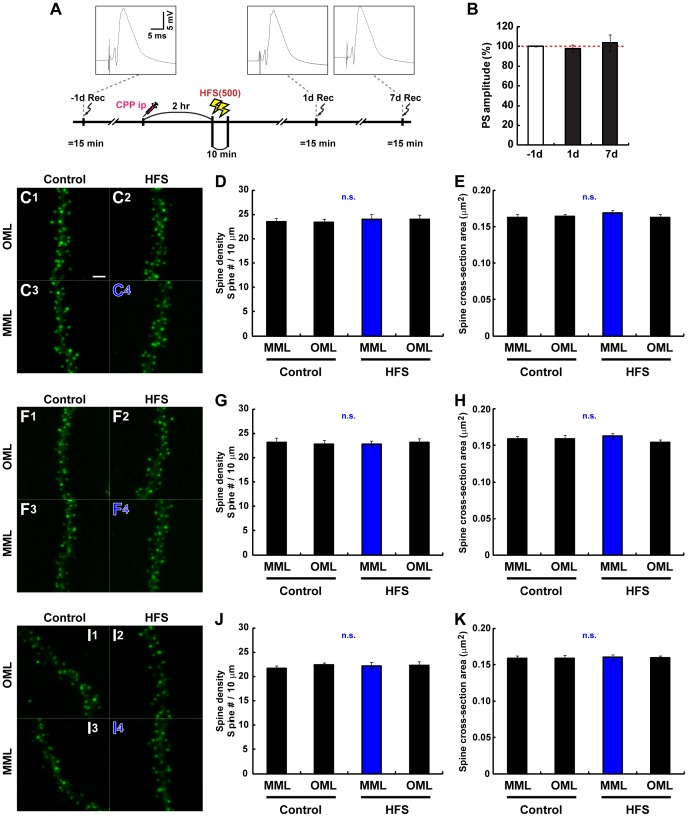
NMDAR activity during HFS is required for LTP-mediated changes in the spinogenesis of new neurons. (**A**) Experimental schedule for CPP pretreatment. Pretreatment with CPP i.p. blocks LTP of PS amplitude in the DG, as previously described (Kitamura *et al*. 2009). Insets are samples of evoked field potential traces recorded at −1, 1, and 7 days post-HFS. (**B**) PS amplitude of rats used in this study at 1 day before, 1 day after, and 7 days after the HFS(500) delivery (−1 d, 1 d, and 7 d, respectively). CPP (10 mg/kg) was injected i.p. 2 h before the initiation of HFS delivery. (**C**) At 12 dpi, CPP was injected i.p. 2 h before HFS(500). Representative z-stack images of dendritic segments of new neurons at 28 dpi in control (**C1, C3**) and HFS-treated hemispheres (**C2, C4**) with CPP i.p. administration. (**C1, 2**) and (**C3, 4**) represent micrographs of OML and MML, respectively. Scale bars for (**C**), (**F**), and (**I**), 2 µm. (**F**) At 16 dpi, CPP was injected i.p. 2 h before HFS(500). Representative z-stack images of dendritic segments of new neurons at 28 dpi in control (**F1, F3**) and of HFS-treated hemispheres (**F2, F4**) with CPP i.p. administration. (**F1, 2**) and (**F3, 4**) represent micrographs of OML and MML, respectively. (**I**) At 21 dpi, CPP was injected i.p. 2 h before HFS(500). Representative z-stack images of dendritic segments of new neurons at 28 dpi observed in control (**I1, 3**) and HFS-treated hemispheres (**I2, 4**) with CPP i.p. administration. (**I1, 2**) and (**I3, 4**) represent micrographs of OML and MML, respectively. Dendritic segments within the HFS-delivered layer are indicated by blue characters (**C4, F4, and I4**). (**D**), (**G**), (**J**) Spine number per 10-µm dendritic fragment in each layer is graphed. (**E**), (**H**), (**K**) Averages cross-sectional area of spines is indicated. (**D, E** *)* Data from new neurons treated with CPP and HFS delivery at 12 dpi. Dendritic fragments for spine analyses: control hemisphere, n = 19, HFS hemisphere, n = 19 from 3 animals. (**G, H**) Data from new neurons treated with CPP and HFS delivery at 16 dpi. Dendritic fragments for spine analyses: control hemisphere, n = 14, HFS hemisphere, n = 17 from 3 animals. (**J, K**) Data from new neurons treated with CPP and HFS delivery at 21 dpi. Dendritic fragments for spine analyses: control hemisphere, n = 19, HFS hemisphere, n = 19 from 3 animals. Data from the HFS-delivered layer are indicated by blue color in each graph. n.s. indicates no significant variance.

For quantitative measurements of spine density and cross-sectional area, analyses were performed with neurons that were relatively isolated from neighboring GFP+ neurons and had untruncated dendrites from MML through OML as Figure 1C. Images of GFP-actin signal were acquired from a clearly extending dendritic fragment of individual cell without intersecting with other GFP+ dendrites and at the center of middle third and outer third of ML under the condition of 0.7-µm intervals using a Plan-Apochromat 63×/1.4 oil lens and digital zooms of ×10 and ×7 with LSM510 (for Figures 1, 2, 3, 4) and LSM700 (for Figure 5) laser-scanning confocal microscopes, respectively (Carl Zeiss, Jena, Germany). Z-stack images were created by LSM image browser or ZEN 2009 software with maximum intensity projections of confocal z-series (Carl Zeiss, Jena, Germany). To define spine density, the number of spines was counted manually, and the length of dendritic segments was determined by tracing the center of the dendritic shaft with the “Distance” function of Metamorph software (Molecular Devices, Downingtown, PA). To define the cross-sectional area of spines, each z-stack image was binarized by appropriate thresholding to bring out the strong edges while minimizing noises and false edges [30]. The threshold for each images were set to be almost same average background pixel number. The edge of each spine was manually traced in Metamorph software (Molecular Devices, Downingtown, PA), and then the area was determined in pixel numbers with the Metamorph “Area” function. The cross-sectional area of each spine was calculated based on the obtained number of pixels and the reduced scale.

For quantitative measurements of BrdU^+^ and BrdU^+^Zif268^+^ cells in the dorsal hippocampus, all BrdU^+^ cells, regardless of size or shape, were counted using a 40× objective (BX41, OLYMPUS), as described previously [29]. A cell was counted if it adjoined the subgranular zone (SGZ) or was positioned in the SGZ or GC layer (GCL) (excluding the hilus region), as described previously [29], [31], [32].

All statistical analyses were performed using StatView Software (Abacus Concepts, Berkeley, CA). Comparisons between two-group data were analyzed by Student’s t-test (two tailed). If the data did not meet the assumptions of the t-test, the data were analyzed using the Mann-Whitney U-test (two tailed). Multiple-group comparisons were assessed using a one-way analysis of variance (ANOVA), followed by the post-hoc Scheffe’s test when significant main effects were detected. If the data met the assumptions, they were analyzed using the post-hoc Fisher’s test. The null hypothesis was rejected at the *P*<0.05 level. Quantitative data in this study are shown as mean ± SEM.

## Results

### Spinogenesis of New Neurons for the First 4 Weeks after Birth in the Rat Adult DG

Because the actin filament (F-actin) is the major cytoskeletal structure in dendritic spines [33], [34], GFP-β-actin-RV provides a clear image of the spines of newly born neurons. We constructed a compatible system of spine labeling of newly born neurons in DG of adult rats by GFP-β-actin-RV infection and *in vivo* LTP induction. To observe spine development, newly born neurons were retrovirally labeled with GFP-β-actin (Figure 1C). GFP-actin allows visualization of spines without altering their morphology [34], [35] and enters all spines [36]. At 12 days post-infection (dpi), no spines were observed, and the distal tips of dendrites did not expand beyond the middle part of the ML in all cells observed (n = 17 neurons from 2 animals) (Figure 1C, panels *a* and *d*, and Figure 1F). Few spines were found at 16 dpi (spine density: 3.93±0.44/10 µm from 2 animals, n = 26 dendritic fragments) (Figure 1C, panels b, e, and f, and Figure 1F), similar to previous reports in mice [11] and rats [37]. Spinogenesis dramatically progressed from 16 to 18 dpi (spine density at 18 dpi: 14.74±0.95/10 µm, n = 29 dendritic fragments from 2 animals; 16 vs. 18 dpi, *P*<0.001, Mann-Whitney U-test) (Figure 1C, panels c, g, and h, and Figure 1F). Moreover, the first dramatic progression of spinogenesis nearly reached plateau by 3–4 weeks post-infection (Figure 1C, panels i and j, and Figure 1F), showing interaction with presynaptic terminals (Figure 1D). These observations were similar to previous reports in mice [11], [22]. Moreover, immunoelectron microscopy revealed that about 80% of GFP-actin-positive spines on 28 dpi neurons contained postsynaptic density and contacted with synaptic vesicles containing structure (78.1±1.9%, n = 3 neurons, total 90 GFP+ protrusions) (Figure 1E, panels a and b). Other protrusions did not satisfy the criteria of functional synapses (Figure 1E, panel c).

Next, to determine whether spine development reflects responsiveness to excitatory input, LTP-inducible stimulation, HFS(500), was delivered in ipsilateral MPP-GC synapses (Figure 1A) at 12 and 28 dpi, and brains were then dissected 1 h after the stimulation (Figure 1G). The HFS(500) in the MML increased the expression rate of Zif268, a product of an immediate early gene, in the GC layer (GCL) (Figure 1H) where GC somata localize (Figure 1A, C). Zif268 expression was significantly increased by HFS in GFP-positive neurons at 28 dpi (control hemisphere, 15.55% ±4.73%; ipsilateral ( = LTP) hemisphere, 52.25% ±6.26; *P*<0.01, Student’s t-test) (Figure 1I, J). By contrast, no detectable signals in GFP-positive neurons were observed in either control or ipsilateral hemispheres at 12 dpi (Figure 1I, J). These results indicate that 4-week-old neurons have features of excitatory neurons. In this study, we therefore mainly observed the spine formation pattern of new neurons at 28 dpi to be an indicator of integration pattern, at which time new neurons passed through the first phase of dramatic development.

### Induction of LTP at the No-spine Stage of New Neurons Specifically Enhances their Later Spinogenesis in the MML

New neurons have no, few, or many spines at 12, 16, or 21 dpi, respectively (Figure 1C, F). LTP was induced at MPP-GC synapses at each representative stage of new neurons’ spinogenesis, and, at first, the spine expression pattern was observed at 28 dpi (Figure 2A). First, LTP was induced in the ipsilateral MML at 12 dpi by delivering 500 pulses of HFS (HFS(500)) to the MPP. Potentiation of population spike (PS) amplitude persisted for 16 days (Figure 2B), field excitatory postsynaptic potential (fEPSP) slope was maintained at least for 7 days (Figure S1). LTP induction at the DG *in vivo* is associated with actin cytoskeleton reorganization, including the long-lasting increase of F-actin content within spines [21]. Thus, the layer specificity of the HFS(500) was monitored by the significant increase of F-actin signal in the MML of the HFS(500)-treated hemisphere. F-actin was quantified by histochemical analysis at 28 dpi using phalloidin, a F-actin-specific probe (in the same animals used for spine investigations below, control side, F_(2, 6)_ = 1.22, *P*>0.360, ANOVA; LTP side, *P*<0.002, ANOVA; MML vs. IML, *P*<0.002; vs. OML, *P*<0.016; post-hoc Scheffe’s test) (Figure 2C1, C2, G). Heterosynaptic LTD is formed in LPP synapses by LTP induction in MPP synapses [38]. We observed significant decrease of F-actin signal at IML in LTP side compared with control side (*P*<0.05, Student’s t-test), and it may be mediated by the heterosynaptic depression which is induced by MML LTP, similarly with a case of OML. By contrast, presynaptic content revealed by synaptophysin signal in the MML and OML at 28 dpi was unchanged by MML LTP induction (Figure 2D1, D2, H). The spine density of new neurons labeled with GFP-actin in the MML and OML at 28 dpi was similar in the control hemisphere (MML, 20.73±0.78/10 µm; OML, 20.51±0.87/10 µm; *P*>0.858, Student’s t-test) (Figure 2E1, F1, I, J). By contrast, LTP induction in the MML at 12 dpi significantly increased the spine number specifically in the MML at 28 dpi (LTP-induced MML, 23.71±0.92/10 µm; ipsilateral OML, 20.05±0.77/10 µm), compared with non-induced layers (*P*<0.011, ANOVA; LTP-induced MML vs. control MML, *P*<0.014; vs. control OML, *P*<0.001; vs. ipsilateral OML, *P*<0.003; post-hoc Fisher’s test) (Figure 2E, F, I, J). Moreover, significant spine enlargement was observed locally in the LTP-induced layer (cross-sectional area of control MML, 0.220±0.006 µm^2^; control OML, 0.211±0.005 µm^2^; LTP-induced MML, 0.243±0.007 µm^2^; ipsilateral OML, 0.208±0.005 µm^2^, *P*<0.001, ANOVA; LTP-induced MML vs. control MML, P<0.006; vs. control OML, *P*<0.001; vs. ipsilateral OML, *P*<0.001; post-hoc Fisher’s test) (Figure 2E, F, K). These results indicate that LTP induction at 12 dpi locally enhances later spinogenesis in new neurons within the LTP-induced layer by 4 weeks of age, although no spines are present at the time of LTP induction (Figure 1C, F).

We also observed the spine expression pattern at 19 dpi (with LSM700, control hemisphere, n = 15 dendritic fragments, HFS hemisphere, n = 15 dendritic fragments from 3 animals). In this case, no significant difference in spine density were appeared between the LTP-induced layer and other layers (control MML, 13.89±0.76/10 µm; control OML, 13.63±0.68/10 µm; LTP-induced MML, 15.31±0.73/10 µm; ipsilateral OML, 12.25±0.84/10 µm; F_(3, 56)_ = 2.76, *P*>0.050, ANOVA). However, the average spine size of new neurons in the LTP-induced layer was significantly increased compared with both layers of the control DG and the OML of ipsilateral DG at 19 dpi (cross-sectional area of control MML, 0.169±0.007 µm^2^; control OML, 0.171±0.006 µm^2^; LTP-induced MML, 0.190±0.004 µm^2^; ipsilateral OML, 0.171±0.006 µm^2^, *P*<0.049, ANOVA; LTP-induced MML vs. control MML, *P*<0.016; vs. control OML, *P*<0.024; vs. ipsilateral OML, *P*<0.030; post-hoc Fisher’s test). These results indicate that maturation of the spines has been locally enhanced by 1 week after LTP induction at 12 dpi although the spine expression rate has not been accelerated.

### MML LTP Induction at the Time of Initial Spinogenesis of New Neurons Inhibits their Later Spinogenesis Specifically in the MML

Next, to investigate the effect of LTP induction at the initial time of spine development on the later spine expression pattern of new neurons, MML LTP was induced at 16 dpi (Figure 3A, B). Specific delivery of the HFS(500) at MML synapses was confirmed by the enhancement of F-actin signal in the MML of ipsilateral DG at 28 dpi (in the same animals used for spine investigations below, control hemisphere, F_(2, 6)_ = 0.71, *P*>0.529, ANOVA; LTP-induced hemisphere, *P*<0.010, ANOVA; MML vs. IML, *P*<0.004; vs. OML, *P*<0.06; post-hoc Fisher’s test) (Figure 3C1, C2, G). No changes in presynaptic content were observed in LTP-induced and non-induced layers at 28 dpi (Figure 3D1, D2, H). In contrast to LTP induction at 12 dpi, no differences in spine size at 28 dpi were observed between the LTP-induced layer and the other layers (cross-sectional area of control MML, 0.214±0.004 µm^2^; control OML, 0.210±0.007 µm^2^; LTP-induced MML, 0.216±0.009 µm^2^; ipsilateral OML, 0.214±0.005 µm^2^; F_(3, 68)_ = 0.13, *P*>0.944, ANOVA) (Figure 3E, F, K). Surprisingly, spine density of new neurons in the LTP-induced layer was drastically decreased compared with both layers of the control DG and OML of the ipsilateral DG at 28 dpi (control MML, 22.49±1.10/10 µm; control OML, 21.19±0.80/10 µm; LTP-induced MML, 14.91±1.05/10 µm; ipsilateral OML, 21.72±0.92/10 µm, *P*<0.001, ANOVA; LTP-induced MML vs. control MML, *P*<0.001; vs. control OML, *P*<0.001; vs. ipsilateral OML, *P*<0.001; post-hoc Scheffe’s test) (Figure 3E, F, I, J).

In addition, we also observed the spine expression pattern at 23 dpi, timing 1 week after the LTP induction (with LSM700, control hemisphere, n = 15 dendritic fragments, HFS hemisphere, n = 14 dendritic fragments from 2 animals). Spine density of new neurons in the LTP-induced layer had been already decreased compared with other layers at 23 dpi (control MML, 18.67±0.81/10 µm; control OML, 18.03±0.66/10 µm; LTP-induced MML, 13.75±0.86/10 µm; ipsilateral OML, 18.05±0.64/10 µm, *P*<0.001, ANOVA; LTP-induced MML vs. control MML, *P*<0.001; vs. control OML, *P*<0.003; vs. ipsilateral OML, *P*<0.003; post-hoc Scheffe’s test) without change in spine size (cross-sectional area of control MML, 0.147±0.004 µm^2^; control OML, 0.149±0.003 µm^2^; LTP-induced MML, 0.144±0.004 µm^2^; ipsilateral OML, 0.142±0.003 µm^2^; F_(3, 54)_ = 0.76, *P*>0.520, ANOVA). This tendency of spine expression pattern was comparable to the results at 28 dpi. These results suggest that LTP induction at 16 dpi locally inhibits the spine expression from early timing after the induction and maintains at least about 2 weeks in an LTP-induced layer-specific manner.

### Induction of MML LTP after the Drastic Spinogenesis Stage of New Neurons Results in MML-specific Spine Enlargement

Next, MML LTP was induced at 21 dpi by delivery of HFS(500) (Figure 4A). Potentiation of PS amplitudes and fEPSP slopes were maintained at 28 dpi (Figure 4B, Figure S1), and the increased F-actin content in the MML of HFS(500)-treated DG was confirmed (in the same animals used for spine investigations below, control hemisphere, F_(2, 6)_ = 4.02, *P*>0.078, ANOVA; LTP-induced hemisphere, *P*<0.002, ANOVA; MML vs. IML, *P*<0.001; vs. OML, *P*<0.03; post-hoc Fisher’s test) (Figure 4C1, C2, G). No change in the expression pattern of synaptophysin was observed at 28 dpi (Figure 4D1, D2, H). In contrast to LTP induction at 12 and 16 dpi, no changes in spine density were noted between the LTP-induced layer and other layers (control MML, 20.40±0.76/10 µm; control OML, 21.12±0.95/10 µm; LTP-induced MML, 22.26±1.03/10 µm; ipsilateral OML, 21.68±0.81/10 µm; F_(3, 60)_ = 0.78, *P*>0.507, ANOVA) (Figure 4E, F, I, J). However, the average spine size of new neurons in the LTP-induced layer was significantly increased compared with both layers of the control DG and the OML of ipsilateral DG at 28 dpi (cross-sectional area of control MML, 0.212±0.006 µm^2^; control OML, 0.204±0.006 µm^2^; LTP-induced MML, 0.241±0.007 µm^2^; ipsilateral OML, 0.191±0.004 µm^2^, *P*<0.001, ANOVA; LTP-induced MML vs. control MML, *P*<0.008; vs. control OML, *P*<0.001; vs. ipsilateral OML, *P*<0.001; post-hoc Scheffe’s test) (Figure 4E, F, K). These results indicate that LTP induction at 21 dpi induces spine enlargement specifically in the LTP-induced layer. This observation is similar to the responses of spines of mature CA1 neurons following LTP induction [39], [40].

We also observed the spine expression pattern at 35 dpi, timing at 1 week later from the first observation (with LSM700, control hemisphere, n = 13 dendritic fragments, HFS hemisphere, n = 14 dendritic fragments from 2 animals). Although significant increase of the spine size in the LTP-induced layer was observed at 28 dpi, the difference of average spine size of new neurons disappeared at 35 dpi (cross-sectional area of control MML, 0.156±0.005 µm^2^; control OML, 0.149±0.004 µm^2^; LTP-induced MML, 0.163±0.004 µm^2^; ipsilateral OML, 0.146±0.004 µm^2^, *P*<0.039, ANOVA; LTP-induced MML vs. control MML, *P* = 0.754; vs. control OML, *P* = 0.192; vs. ipsilateral OML, *P* = 0.067; post-hoc Scheffe’s test). Moreover, spine density of new neurons in the LTP-induced layer was decreased compared with both layers of the control DG and OML of the ipsilateral DG during the period from 28 to 35 dpi (control MML, 26.44±1.28/10 µm; control OML, 25.96±1.01/10 µm; LTP-induced MML, 20.65±0.97/10 µm; ipsilateral OML, 25.18±0.94/10 µm, *P*<0.001, ANOVA; LTP-induced MML vs. control MML, *P*<0.004; vs. control OML, *P*<0.010; vs. ipsilateral OML, *P*<0.031; post-hoc Scheffe’s test). These data suggest that integration of new neurons into circuit is locally and transiently enhanced by LTP induction at 21 dpi, but the enhancement is erased by 2 weeks after the induction.

### NMDAR Activity during the Delivery of HFS to MPPs is Required for LTP Induction and all Changes in the Spine Expression Pattern of New Neurons

Because NMDA receptors (NMDARs) play a crucial role in memory formation and long-lasting neural plasticity including LTP [17], we finally investigated whether the changes in spine expression pattern of new neurons are mediated by NMDAR activity during LTP induction. The selective NMDAR antagonist 3-(2-carboxypiperazin-4-yl)-propyl-1-phosphonic acid (CPP) was used to block NMDAR. CPP (10 mg/kg) was intraperitoneally (i.p.) injected 2 h prior to HFS(500) (Figure 5A) at 12, 16, or 21 dpi, and the spine expression pattern of new neurons was then observed at 28 dpi. CPP application with this protocol clearly blocked LTP induction in DG (Figure 5B), as described previously [29], [41]. Similarly, F-actin signal intensity at 28 dpi within the ipsilateral DG MML showed no change compared with other layers after HFS delivery at 12, 16, or 21 dpi (HFS at 12 dpi, control hemisphere, F_(2, 6)_ = 0.04, *P*>0.963, ANOVA; LTP hemisphere, F_(2, 6)_ = 0.25, *P*>0.783, ANOVA; HFS at 16 dpi, control hemisphere, F_(2, 6)_ = 0.16, *P*>0.854, ANOVA; LTP hemisphere, F_(2, 6)_ = 0.76, *P*>0.508, ANOVA; HFS at 21 dpi, control hemisphere, F_(2, 6)_ = 1.16, *P*>0.376, ANOVA; LTP hemisphere, F_(2, 6)_ = 0.26, *P*>0.777, ANOVA) (Figure S2A–F). No significant differences in spine density (F_(3, 72)_ = 0.15, *P*>0.927, ANOVA) (Figure 5C, D) or spine size (F_(3, 72)_ = 0.56, *P*>0.641, ANOVA) (Figure 5C, E) were observed at 28 dpi after treatment with HFS(500) + CPP at 12 dpi. The suppression of spine expression observed in LTP induced at 16 dpi (Figure 3I, J) was completely blocked (F_(3, 58)_ = 0.10, *P*>0.961, ANOVA) (Figure 5F, G) with no change in spine size (F_(3, 58)_ = 0.77, *P*>0.514, ANOVA) (Figure 5F, H) when the HFS was delivered at 16 dpi with CPP application. Likewise, spine expression patterns were comparable between the LTP-induced layer and other layers at 28 dpi when HFS(500) was delivered in the presence of CPP at 21 dpi (spine density, F_(3, 72)_ = 0.31, *P*>0.817, ANOVA; spine cross-sectional area, F_(3, 72)_ = 0.03, *P*>0.991, ANOVA) (Figure 5I, J, K). Therefore, the differential regulation of later spine expression pattern observed in this study was mediated by the activity of NMDAR at the time of HFS delivery.

### Induction of LTP in New Neurons Approximately 12 Days in Age Enhances their Later Functional Integration into Preexisting Circuits

**Figure 6:**
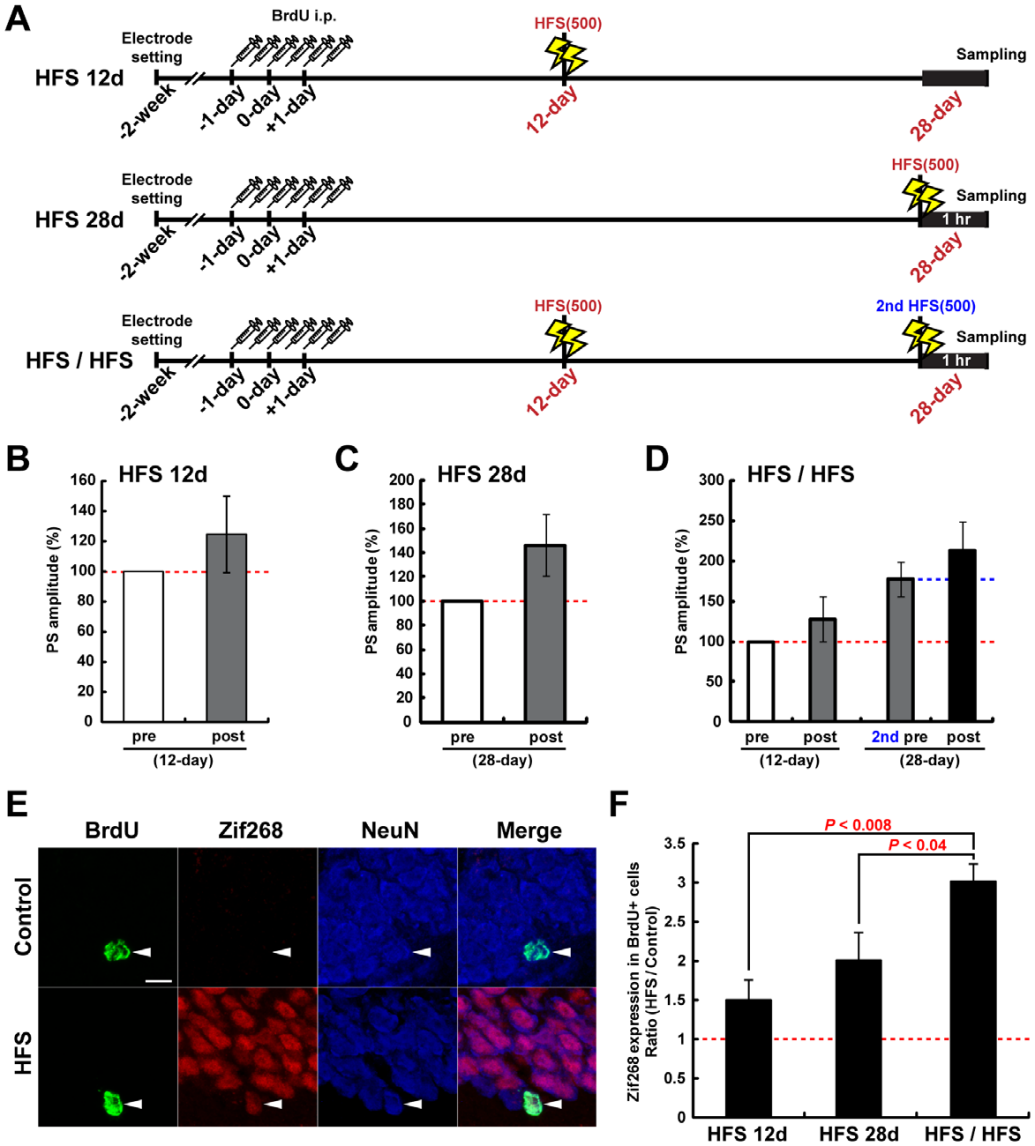
Functional integration of new neurons is enhanced by MML-LTP induction at 12 days of age. (**A**) Experimental schedules (**B, C**) PS amplitudes of the DG obtained from rats under the “HFS 12d” and “HFS 28d” conditions are shown in panels **B** and **C**, respectively. Pre- and post-HFS delivery are indicated by “pre” and “post”, respectively. (**D**) PS amplitudes of the DG obtained from rats under the “HFS/HFS” condition. Both HFS(500) at day 12 and day 28 increased PS amplitude. (**E**) Representative z-stack images of BrdU^+^ cells of the control hemisphere and HFS(500)-induced hemisphere in the “HFS/HFS” condition. Signals of BrdU, Zif268, and NeuN are shown as green, red, and blue, respectively. The upper side and lower side of each image are the molecular layer and the hilus region, respectively. Arrowheads in each panel indicate the nucleus of the same BrdU^+^ cell. Scale bar, 10 µm. (**F**) HFS(500)-delivered hemisphere to control the hemisphere ratio (HFS/Control) of Zif268 expression in BrdU^+^ cells. The data were obtained from three animals in each condition. *P* values from post-hoc Fisher’s test are shown in the graph.

To evaluate functional integration of the 4-week-old new neurons that underwent LTP induction 16 days prior, we investigated the rate of circuit activity-dependent Zif268 expression in BrdU^+^ cells. We established three experimental groups that were injected with BrdU for 3 consecutive days: “HFS 12d” and “HFS 28d”, in which LTP was induced 12 or 28 days after the second day of BrdU injection, respectively (Figure 6A, B, C), and “HFS/HFS”, in which LTP was induced at 12 and 28 days after the second day of BrdU injection (Figure 6A, D).

BrdU is incorporated into the newly synthesized DNA of dividing cells. The majority of 4-week-old BrdU^+^ cells that were localized in or attached to the GCL express NeuN, a marker of mature neurons [29], [31], [32]. All BrdU^+^Zif268^+^ cells were NeuN^+^ (Figure 6E). We mainly focused on differences in the Zif268 induction ratio between the ipsilateral and control hemispheres of each animal. In “HFS 12d”, no significant difference in the Zif268 expression rate of 4-week-old BrdU^+^ cells was observed between control and ipsilateral DGs (control side, 11.3% ±3.3; ipsilateral side, 15.2% ±2.9, *P*>0.42, Student’s t-test). By contrast, in the “HFS 28d” and “HFS/HFS” groups, LTP induction at day 28 significantly enhanced Zif268 expression in 4-week-old cells (HFS 28d: control side, 12.3% ±1.5; ipsilateral side, 23.6% ±1.3, *P*<0.005, Student’s t-test; HFS/HFS: control side, 9.3% ±1.1; ipsilateral side, 28.4% ±2.3, *P*<0.002, Student’s t-test). The Zif268 induction ratio in 4-week-old cells significantly increased in the “HFS/HFS” group compared with the other conditions (ipsilateral side/control side: HFS 12d, 1.5±0.4; HFS 28d, 2.0±0.4; HFS/HFS, 3.0±0.2, *P*<0.02, ANOVA; HFS 12d vs. HFS/HFS, *P*<0.008; HFS 28d vs. HFS/HFS, *P*<0.04; post-hoc Fisher’s test) (Figure 6F). HFS(500) induced at any time elicits no change in the ratio of neuronal differentiation in 4-week-old BrdU^+^ cells [29], [31]. In addition to the spine expression pattern results shown in Figure 2, these previous findings and our data strongly suggest that when LTP is induced in neurons at approximately 12 days of age, their later functional integration into activated circuits is enhanced.

## Discussion

### Developmental Stages of Newly Born Neurons

In the hippocampal DG, new neurons are continually generated and integrated into preexisting circuits throughout adulthood. The spine expression pattern is closely correlated with the excitatory postsynaptic response of developing newly born neurons in adult DG [11]–[14]. Our observations of the dendritic morphogenesis of newly born neurons labeled by RV-mediated expression of GFP-actin identified several developmental stages of spine formation in adult rats. The initial timing of spinogenesis occurred in new neurons aged at ∼16 days (Figure 1C, F), which is consistent with previous reports both in mice [11] and rats [37]. In the DG of adult mice, most dendritic protrusions of new neurons labeled by RV are formed between 21 and 30 dpi [11], [22]. However, our results indicated that spinogenesis increased sharply at around 18 dpi (Figure 1C, F). Thus, the dramatic progression of spine growth occurs about 1 week earlier in rat DG than mouse DG. New neurons in adult rats have been reported to show a mature neuronal marker profile by 4 weeks after birth, which is 1–2 weeks earlier than mice [32]. Immunoelectron microscopy revealed that about 80% of spines on 28 dpi neurons meet structural requirements for excitatory synapse (Figure 1E). Accordingly, the date of our observation, 28 dpi, is appropriate for investigating the effects of earlier experiences during developmental on the spine patterns of mature new neurons in adult rat. In adult mice, gradual development of spines and lower LTP induction threshold are still observed in new neurons up to 180 days and 6–8 weeks of age, respectively [11], [42]. Thus, new neurons pass through several developmental stages, during which each neuron could be differentially integrated into preexisting circuits in an experience-dependent manner.

### Effects of LTP at 12 dpi on Later Spine Formation Pattern

**Figure 7:**
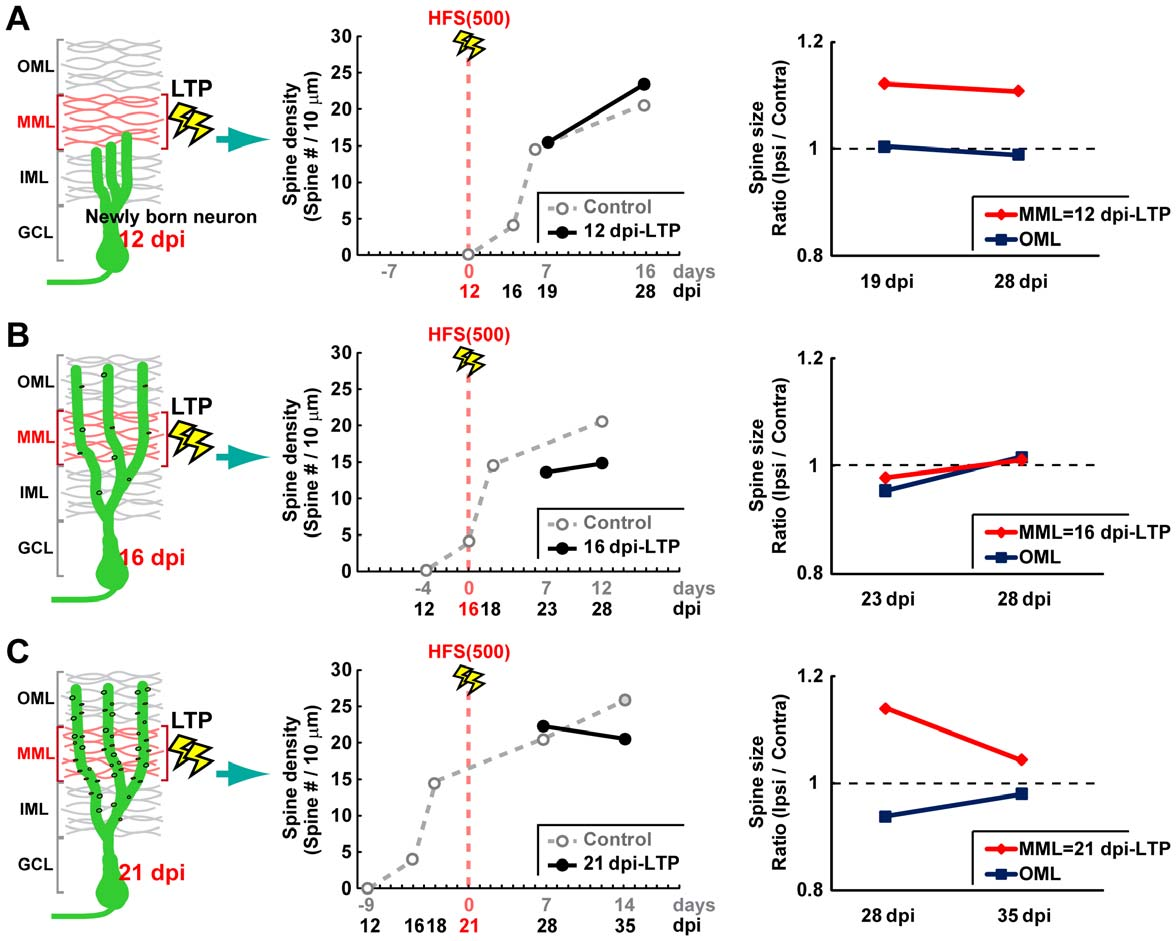
Formation of neural plasticity differentially regulates the integration of new neurons into the activated circuit. (**A**) LTP induction at the no-spine stage of new neurons (12 dpi) specifically increases their spine size and spine expression rate within the LTP-induced layer by 3 (19 dpi) and 4 weeks of neuronal age (28 dpi), respectively. (**B**) LTP induction at the time of initial spinogenesis of new neurons (16 dpi) locally inhibits the later expression of spines in an LTP-induced layer-specific manner. (**C**) LTP induction after the drastic spinogenesis stage of new neurons, at 21 dpi, induced their later spine enlargement in the LTP-induced layer at 28 dpi. The spine enlargement is return to basal level, and the later spinogenesis is inhibited by 35 dpi. All LTP-mediated changes are tightly correlated with actual formation of the activity-dependent neural plasticity. **Left,** Schematic representation of morphology of each new neurons at 12, 16, or 21 days after birth ( = 12, 16, or 21 dpi, respectively). High frequency stimulation [HFS(500)] for LTP induction is specifically delivered at MML of ipsilateral hemisphere in all experiments of present study. **Middle,** The mean values of spine density at indicated timing under control and LTP-induced conditions are plotted by white (represented in Figure 1F) and black circles, respectively. **Right,** Graph shows the mean values of ipsilateral (Ipsi)/contralateral (Contra) ratio of spine cross-sectional area in MML and OML. Abbreviations: GCL, granule cell layer; IML, MML, and OML, inner, middle, and outer molecular layer, respectively.

Interestingly, we observed that LTP induction at 12 dpi specifically increased spine expression rate and spine size within the LTP-induced layer by 4 weeks but not 3 weeks of neuronal age (Figures 2, 7A), although no spines were present at the time of LTP induction (Figure 1C, F). This observation is quite similar to the effect of HP-dependent learning in new neurons at the second week of age [15]. Thus, these new neurons seem to be preferentially integrated later into neural circuits in which synaptic plasticity had been induced (Figure 7A).

How does LTP induction at 12 dpi influence later spine expression pattern when 12-dpi neurons have no spines? Spatial learning facilitates the long-term survival (>1 month) of 2-week-old new neurons in mice [43]. The surviving new neurons have also been suggested to preferentially incorporate into the neural circuits that encode the learned behavioral experiences [43]. Therefore, LTP induction and experience of spatial learning may similarly tag some molecular traces that are required for changes in synaptic morphology and physiology and would later enhance the spine formation of new neurons. Some candidate molecular traces are found in extracellular matrix. For example, hippocampal matrix metalloproteinase-3 (MMP-3) and -9 (MMP-9) are transiently increased during hippocampal-dependent learning in an NMDAR activity-dependent manner, and inhibition of their activity prevents LTP and hippocampal-dependent learning [44]. Neural cell adhesion molecule (NCAM) has been implicated in synaptic plasticity including LTP and learning and memory [45]. DG LTP induction in the perforant path (PP) synapses increases NCAM expression at the spine synapses of GCs in the LTP-induced layer [45]. In contrast, HP progenitors and new neurons initially receive depolarizing GABAergic input before glutamatergic synapses are established. Therefore, mechanisms for activity-dependent regulation of adult neurogenesis may sense neuronal network activity through local ambient GABA levels before forming glutamatergic synapses. However, inhibition of GABA_A_ receptor from 14–27 dpi does not exert significant effects on the spine density or dendritic length of 28-day-old new neurons in mice [46]. This finding suggests a minor role of GABAergic signaling in the maturation process of new neurons after their second week. Alternatively, although 12-dpi neurons have no spines, about 30% of 12-day old neurons in adult rats receive glutamatergic inputs in an *in vitro* slice preparation [47], which may lead to subsequent spine growth. Future studies are needed to identify and clarify the mechanisms underlying later enhancement of spinogenesis in new neurons.

What is the functional relevance of the enhanced later integration of new neurons into LTP-induced circuits? Spine density possibly correlates with the available postsynaptic capacity. Repeated delivery of HFS to PP synapses results in impaired spatial learning in rats [48], suggesting strongly that the HP capacity for information processing is saturated by repeated HFS. Therefore, HFS(500) applied to the MPP in this study may decrease postsynaptic capacity of preexisting circuits in the LTP-induced layer. Given this situation, continuous and increased later integration of new neurons after LTP induction possibly compensates for the decreased processing ability for new information within the LTP-induced layer. Our present data suggest that later functional integration is specifically enhanced in activated circuits when LTP is induced in new neurons at approximately 12 day of age (Figures 2, 6). In addition to the increase in spine density and enhancement of functional integration, it is predicted that new neurons willingly process the new input based on their features of increased excitability and lower LTP induction threshold [42], [49]. Indeed, spine size had been already enlarged in LTP induced layer 1 week after induction whereas the spine density was not changed between control and LTP-induced layer (Figure 7A). Enlarged spines are implicated as sites of acquisition of information, because of correlation between spine volume and number of functional AMPA receptors [39], [40].

Alternatively, the enhanced integration of new neurons after LTP induction may promote the gradual decay of DG LTP. DG LTP is reversed by exposure of rats to an enriched environment after LTP induction [50]. Blockade of NMDAR activity after LTP induction leads to slower decay of DG LTP [51]. Both enriched environment and NMDAR activity positively regulate adult neurogenesis [52]–[54]. In addition, the inhibition of neurogenesis in DG sustains LTP of the fEPSP slope [4]. Integration of new neurons has been suggested to have an impact on the wiring pattern of preexisting circuits [55] due to the likelihood of a competitive situation between new neurons and preexisting GCs [22]. We did not observe an increase in presynaptic content, as measured by synaptophysin labeling, within the LTP-induced layer (Figure 2D, H). Finally, LTP of the fEPSP slope returns to basal levels 2 weeks after induction (Figure S1) [4]. Taken together, a likely scenario can been imagined in which new protrusions from new neurons actively invade into LTP-induced preexisting synapses more frequently than normal (non-LTP) synapses at the timing of initial spinogenesis at around 16–18 dpi, several days after the LTP induction. These protrusions would then synapse with presynaptic boutons that have already made synaptic contact with spines of mature neurons, gradually enlarge, and finally win against the older spines, leading to the gradual LTP decay. Thus, time-lagged spinogenesis of new neurons may play an active role in specific and positive renewal of the DG circuits that have acquired and stored old information. The time courses of the maturation and functional integration of new neurons and the progressive decay of the hippocampal dependency of hippocampus-dependent memory are quite similar (∼1 month) in rodents. The decay of the memory process in hippocampus is positively regulated by adult neurogenesis in mice [4]. In monkeys, the maturation time of new neurons is approximately 6 times longer than that in rat [56], and the retention time of memories in the hippocampus is approximately 3 months [57]. The correlation of time windows between the processes of adult neurogenesis and the hippocampal dependency of acquired memories in rodents and monkeys may suggest that adult neurogenesis in primates selectively participates in information clearance from preexisting circuits of the dentate gyrus, even if the rate of adult neurogenesis is lower than rodents [1].

### Effects of LTP at 16 dpi on Later Spine Formation Pattern

LTP induction at 16 dpi inhibited later spine expression specifically within the LTP-induced layer (Figures 3, 7B). To our knowledge, this result is the first report of the negative regulation of spine expression by neural activity. Thus, the question arises as to how LTP induction at 12 dpi and 16 dpi direct opposite patterns in spine expression at 28 dpi. Differences in developmental stage may underlie this effect. A sharp increase in spine expression occurs at 16 dpi (Figure 1C, F). LTP induction should increase the spine volume of mature neurons [58], and the enlarged state may persist for at least 24 h in the DG [41]. In this situation, synaptic contact at the LTP-induced synapses of mature neurons should be tight immediately following LTP induction, and there may be no space for filopodia budding by new neurons. Indeed, electron microscopy analysis suggests that new neurons in the adult DG compete with existing GCs at excitatory synapses [22]. Thus, competitive bias from the mature neurons at activated synapses inhibits the later spine expression of new neurons. The 12-dpi neurons begin to protrude spines several days after LTP induction. DG LTP of the fEPSP slope elicited by HFS(500) decays gradually, and the magnitude of LTP at 1 week is about half the initial magnitude [4]. Therefore, this competitive bias does not inhibit the spinogenesis of the 12-dpi neurons.

### Effects of LTP at 21 dpi on Later Spine Formation Pattern

The neurons in which LTP was induced at 21 dpi passed the period of sharply increased spinogenesis, and these neurons had a number of spines on their dendritic shafts (Figure 1C, F). In rats, the expression of neural activity marker Zif268 is detectable in new neurons from day 16 of age in a LTP induction-dependent manner [31]. Thus, the 21-dpi neurons were already functionally integrated into preexisting DG circuits at PP synapses. LTP induction at 21 dpi induced only spine enlargement of new neurons in the LTP-induced layer (Figures 4, 7C). DG LTP is associated with an increase in spine volume without any change in spine density on GCs [58]. Thus, the 21-dpi and preexisting mature neurons show a quite similar response to LTP induction. Actually, the spine enlargement returned to control level by 2 weeks after LTP induction (Figure 7C), and the time course is quite similar with decay of LTP of the fEPSP slope (Figure S1) [4].

By contrast, spine size in the OML ipsilateral to the LTP-induced DG showed tendency to decrease compared with both layers of the contralateral side (Figure 4C, D, and *I*). Long-term depression (LTD) is another type of neural plasticity, and LTD of hippocampal neurons accompanies shrinkage of dendritic spines [59]. Heterosynaptic LTD is formed in LPP synapses by LTP induction in MPP synapses, and this plastic change persists for 2 weeks [38]. Thus, heterosynaptic OML LTD may be coincidently induced in 21-dpi neurons by the MML LTP induction in a similar manner to mature GCs.

### Timing- and Layer-specific Effects

Morris water maze, a HP-dependent spatial learning task, promotes the survival of new neurons approximately 10-days-old and enhances the cell death of younger neurons about 7-days-old [8]. Similarly, LTP induction specifically enhances the survival of new neurons at around 10 days of age, but not at 4 or 20 days of age [29]. We observed LTP timing-dependent bidirectional regulation of the spine formation pattern of new neurons, in which LTP differentially enhanced or suppressed later spine formation, dependent on new neurons’ developmental stages (Figures 2, 3, 4, 7). LTP and HP-dependent learning may selectively add or remove new neurons in the DG according to their maturity by regulating their spine formation pattern. Consequently, the different stages of new neurons’ development could possibly play various roles in information processing by modulating the connectivity of preexisting circuits.

Morris water maze training promotes the later spine expression of new neurons in their second week after birth [15]. However, whether the effect is cell-wide or restricted to certain spines had remained unclear. In the present study, we have shown that MML LTP directs various patterns of later spine expression by new neurons only in the MML. Therefore, LTP does not affect later cell-wide spine expression; instead, it locally influences synapse formation at the restricted area that has received synaptic inputs.

### NMDAR Dependence

NMDAR activity is required for spatial learning and induction of long-term synaptic plasticity at various HP synapses [60] including LTP of PP-GC synapses (Figure 5B) [29], [61]. Blockade of NMDAR activity at the time of LTP induction inhibited all changes in the spine expression of new neurons observed in this study (Figure 6). These results strongly suggest that activity-dependent neural plasticity, but not a simple transient increase in neural activity, in DG circuits is required to alter the later spine expression pattern of new neurons.

In this paper, we addressed the questions of whether the hippocampal LTP induction affects later spine expression patterns of new neurons in an induction-timing-dependent manner, and whether these effects are restricted to the area where LTP is induced. Our results indicate that LTP differentially regulates the integration of new neurons into the activated circuit, dependent on their developmental stage. Consequently, new neurons at different developmental stages may play distinct roles in processing the acquired information by modulating the connectivity of activated circuits via their integration.

## Supporting Information

Figure S1 **Duration of MML LTP monitored by fEPSP slope of rats used in this study.** Pooled data from DG fEPSP obtained from rats used for experiments in Figures 2, 3, and 4 (pre, post, and 1d; n = 9). Data at 7 d, 12 d, and 16 d are from the rats in Figures 4 (n = 3), 3 (n = 3), and 2 (n = 3), respectively.
(TIF)

Figure S2 **CPP pretreatment blocks HFS-mediated F-actin rearrangement in MLs.** (**A–F**) CPP pretreatment 2 h before HFS(500) blocks the rearrangement of F-actin content in MLs. (**A**), (**C**), (**E**), Representative fluorescence micrographs of DG at 28 dpi showing F-actin (red) and nuclear (blue) signals. Left panel, control hemisphere. Right panel, ipsilateral hemisphere, to which HFS(500) was delivered. CPP pretreatment and HFS(500) delivery were carried out at 12 dpi (**A**), 16 dpi (**C**), and 21 dpi (**E**). (**B**), (**D**), (**F**), Graphs show average intensity of F-actin in each DG layer in arbitrary units (AU). (**B**) HFS+CPP at 12 dpi. (**D**) HFS+CPP at 16 dpi. (**F**) HFS+CPP at 21 dpi. Scale bar, 50 µm for (**A**), (**C**), and (**E**). Data from the HFS-delivered layer are indicated by blue color in each graph.
(TIF)
