# Supplementary figures and images for: Spine Formation Pattern of Adult-Born Neurons Is Differentially Modulated by the Induction Timing and Location of Hippocampal Plasticity
Source: PLoS One. 2012 Sep 14;7(9):e45270. doi: 10.1371/journal.pone.0045270 (PMC3443223; doi:10.1371/journal.pone.0045270)

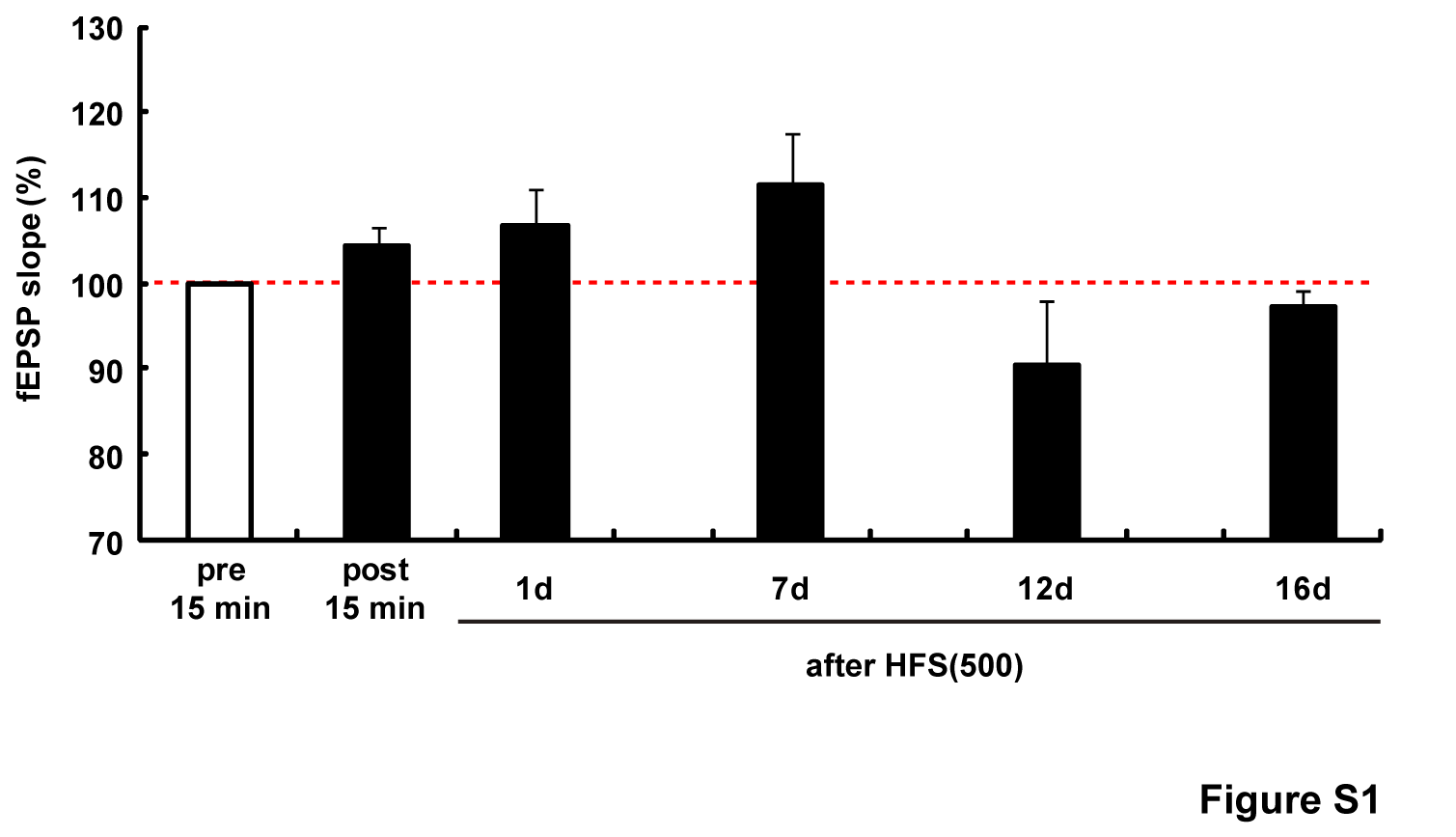

Supplement: Figure S1 — Duration of MML LTP monitored by fEPSP slope of rats used in this study. Pooled data from DG fEPSP obtained from rats used for experiments in Figures 2, 3, and 4 (pre, post, and 1d; n = 9). Data at 7 d, 12 d, and 16 d are from the rats in Figures 4 (n = 3), 3 (n = 3), and 2 (n = 3), respectively. (TIF) [file pone.0045270.s001.tif]

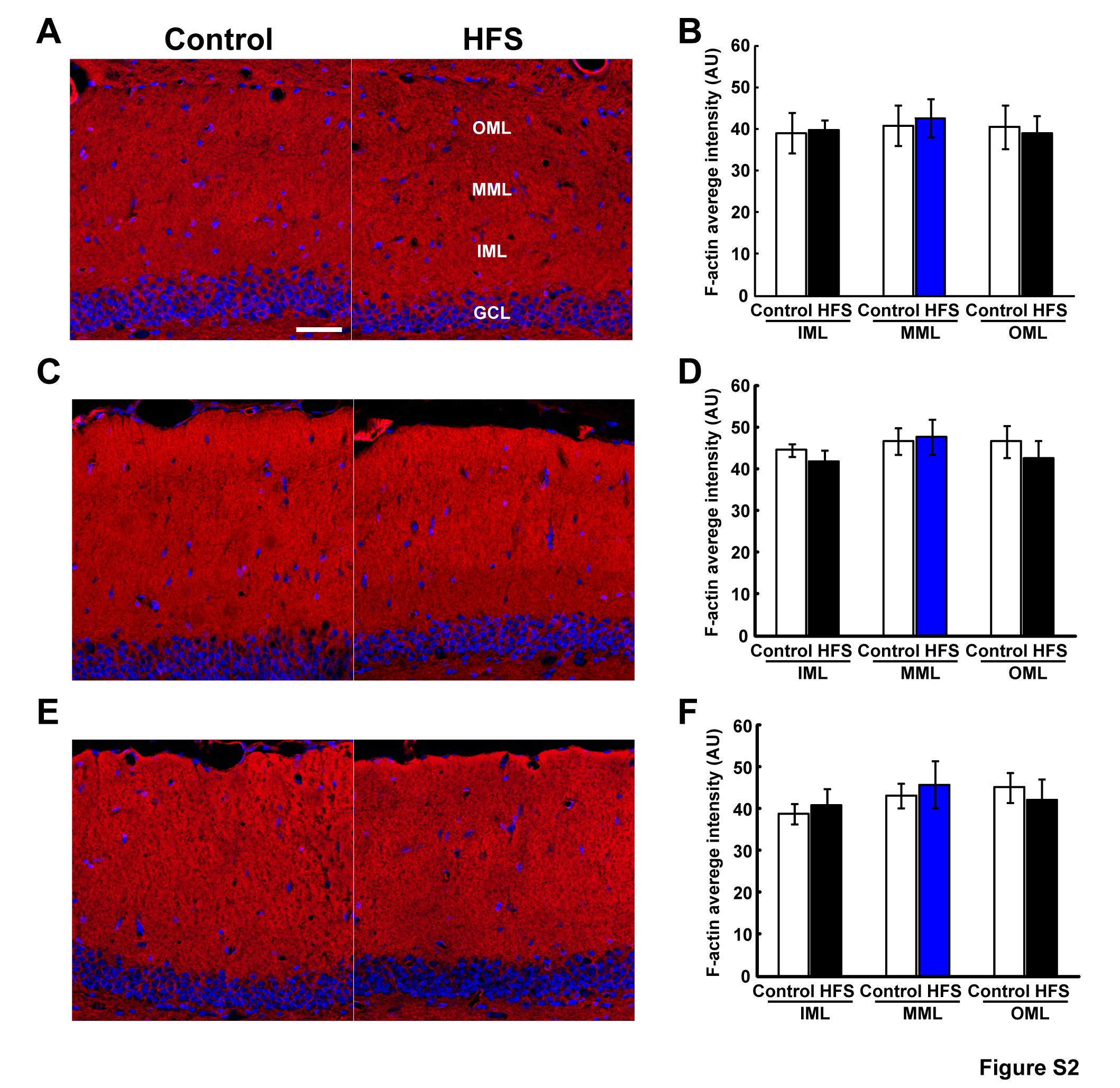

Supplement: Figure S2 — CPP pretreatment blocks HFS-mediated F-actin rearrangement in MLs. (A–F) CPP pretreatment 2 h before HFS(500) blocks the rearrangement of F-actin content in MLs. (A), (C), (E), Representative fluorescence micrographs of DG at 28 dpi showing F-actin (red) and nuclear (blue) signals. Left panel, control hemisphere. Right panel, ipsilateral hemisphere, to which HFS(500) was delivered. CPP pretreatment and HFS(500) delivery were carried out at 12 dpi (A), 16 dpi (C), and 21 dpi (E). (B), (D), (F), Graphs show average intensity of F-actin in each DG layer in arbitrary units (AU). (B) HFS+CPP at 12 dpi. (D) HFS+CPP at 16 dpi. (F) HFS+CPP at 21 dpi. Scale bar, 50 µm for (A), (C), and (E). Data from the HFS-delivered layer are indicated by blue color in each graph. (TIF) [file pone.0045270.s002.tif]
